# Cardiac Allograft Vasculopathy Among Peripartum Cardiomyopathy Heart Transplant Recipients in the United States

**DOI:** 10.1016/j.jacadv.2026.102673

**Published:** 2026-03-18

**Authors:** Juka S. Kim, Alexandra M. Klomhaus, Negeen Shahandeh, Rushi V. Parikh

**Affiliations:** a Division of Medicine, Department of Medicine, Stanford University, Stanford, California, USA; b Department of Medicine Statistics Core, University of California-Los Angeles, Los Angeles, California, USA; c Division of Cardiology, Department of Medicine, University of California-Los Angeles, Los Angeles, California, USA

**Keywords:** cardiac allograft vasculopathy, heart transplantation, peripartum cardiomyopathy

**Figure:**
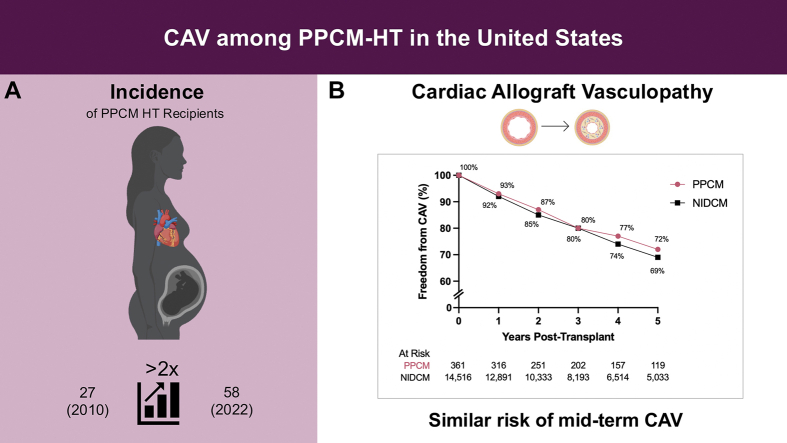

**What is the clinical question being addressed?**

What are the incidence and predictors of cardiac allograft vasculopathy PPCM heart transplant recipients in the contemporary era?

**What is the main finding?**

PPCM heart transplants have more than doubled annually from 27 in 2010 to 58 in 2022. PPCM recipients had a similar risk of developing cardiac allograft vasculopathy at 5 years compared with nonischemic dilated cardiomyopathy recipients.

Peripartum cardiomyopathy (PPCM) is defined as new heart failure with reduced ejection fraction <45% in late pregnancy or soon after delivery in the absence of another identifiable etiology. PPCM is a leading cause of maternal death and contributes to significant morbidity, with 1 to 11% of patients requiring heart transplantation (HT).^1^, ^2^, ^3^ Although PPCM overall remains a rare indication for HT—4% of female HT recipients—the number of women diagnosed with PPCM is growing, owing to the global trend of increasing maternal age and concurrent increase in underlying comorbidities.^1^ Notably, previous studies have consistently shown worse post-HT outcomes, including graft failure and mortality, in this high-risk cohort.^3^^,^^4^

Cardiac allograft vasculopathy (CAV) is an immune- and inflammatory-mediated process of the allograft coronary circulation resulting in diffuse luminal narrowing and ultimately graft failure. PPCM HT recipients are hypothesized to be particularly vulnerable to CAV as pregnancy exposes the maternal immune system to fetal antigens and may cause allosensitization, which can drive chronic endothelial injury.^2^ However, data regarding the association of PPCM with CAV are limited. Therefore, we sought to perform a contemporary analysis of the incidence and predictors of CAV in patients undergoing HT for PPCM in the United States.

## Methods

We performed a retrospective analysis of adult HT recipients with a diagnosis of PPCM or nonischemic dilated cardiomyopathy (NIDCM) using Scientific Registry of Transplant Recipients (SRTR) database from January 1, 2010 to December 31, 2022. Exclusion criteria included retransplantation, an absence of follow-up or angiographic data within 5 years post-HT, and unknown CAV status at the last follow-up or follow-up at which censoring occurred. Similar to prior SRTR-based studies, CAV was defined as any angiographic coronary artery disease.^5^ The primary end point was the development of CAV within 5 years post-HT. This study was deemed exempt by the University of California-Los Angeles Institutional Review Board.

Unadjusted comparisons between cohorts were performed using independent sample t-tests, chi-square tests, or Wilcoxon rank-sum tests, as appropriate. We used a Cox proportional hazards regression model fit to interval-censored data to specifically evaluate associations with time-to-first CAV over the first 5 years post-HT while accounting for differential follow-up time; this model was not designed for prediction or external validation purposes. Proportional hazards assumptions and collinearity were assessed. Twenty-six key recipient, donor, and transplant characteristics were selected as covariates based on clinical relevance and prior work—including our own—in the CAV space.^5^ Covariates were analyzed for their association with CAV using univariable regression, and those with *P* < 0.10 were retained in the multivariable model.

## Results

Of the 14,877 HT recipients, 361 (2%) had PPCM and 14,516 (98%) had NIDCM. Overall, the average age was 51 years and 31% were females. Patients with PPCM were younger (37 vs 51 years, *P* < 0.01), more likely to be Black (55% vs 33%, *P* < 0.01), and had fewer traditional risk factors such as diabetes (15% vs 24%, *P* < 0.01). In addition, PPCM recipients had less cytomegalovirus mismatch (21% vs 26%, *P* = 0.04) and were more likely to receive antithymocyte globulin induction therapy (27% vs 21%; *P* < 0.01), proliferation signal inhibitor (PSI) maintenance therapy (14% vs 11%, *P* = 0.04), and experience acute rejection within 1-year post-HT (58% vs 41%; *P* < 0.01).

**Figure 1:**
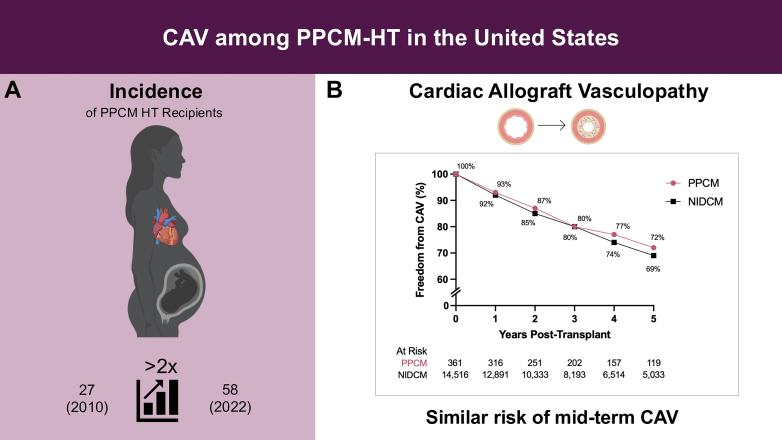
**Outcomes in Peripartum Cardiomyopathy Heart Transplantation Recipients** (A) Schematic illustration demonstrating the incidence of PPCM in the modern HT era and (B) the cumulative freedom from CAV among PPCM vs NIDCM recipients. CAV = cardiac allograft vasculopathy; HT = heart transplantation; NIDCM = nonischemic dilated cardiomyopathy; PPCM = peripartum cardiomyopathy.

The number of annual PPCM transplants rose more than 2-fold from 27 in 2010 to 58 in 2022 (Figure 1A). The incidence of CAV at 5 years was 28% in PPCM and 31% in NIDCM (*P* = 0.46) (Figure 1B).

In multivariable analysis, PPCM recipients had a similar risk of developing CAV within 5 years compared with NIDCM (HR: 0.94; 95% CI: 0.72-1.22; *P* = 0.62). Key factors associated with CAV were Black race (HR: 1.09; 95% CI: 1.00-1.19; *P* = 0.04), PSI use (HR: 1.79; 95% CI: 1.61-2.00, *P* < 0.01), and acute rejection during the first year (HR: 1.23; 95% CI: 1.14-1.33; *P* < 0.01). Among the PPCM subgroup, PSI use (HR: 2.02; 95% CI: 1.14-3.60; *P* = 0.02) and acute rejection (HR: 1.98; 95% CI: 1.15-3.39; *P* = 0.01) were associated with an increased risk of CAV.

## Discussion

In this contemporary national registry-based analysis, the salient findings were: 1) the annual number of PPCM HTs has more than doubled over the past decade, reaching 58 in 2022; 2) PPCM recipients had a similar 5-year risk of CAV compared with NIDCM recipients; and 3) within the PPCM subgroup, those who developed acute rejection during the first year post-HT had a greater risk of CAV.

To the best of our knowledge, prior data on CAV in PPCM are limited to a small study of 69 PPCM recipients from 1990 to 2005 that reported a similar incidence of long-term CAV between PPCM and NIDCM recipients.^4^ Our findings build on these data and suggest that although PPCM may not be a major risk factor for midterm CAV in the modern era of HT, closer monitoring beyond standard CAV surveillance and immunosuppression protocols may be considered for those with early acute rejection. Longer-term follow-up is needed to determine if between-group differences emerge over time.

Our study, however, should be interpreted within the context of important limitations. First, the SRTR database does not capture several key confounders (eg sensitization, parity, prior transfusions, donor-specific antibodies, and cytomegalovirus infection); the inability to account for these variables may have masked an association between PPCM and CAV. Specifically regarding sensitization, data were reported as noncalculated panel reactive antibody before 2015 and as calculated panel reactive antibody after 2015, which precluded an accurate analysis. Other potential confounders included aspirin and statin use, although meaningful differences in their use are unlikely as they are universal to post-HT therapy. Second, the registry does not collect more sensitive intravascular ultrasound data or International Society of Heart and Lung Transplantation CAV grades. This lack of granularity likely resulted in an underestimation of CAV incidence and precluded insights into prognosis, respectively. Lastly, SRTR does not record the exact dates of PSI initiation and CAV detection, which likely resulted in the paradoxical finding that PSI use during the first year post-HT was associated with an increased risk of CAV. PSIs are commonly initiated to mitigate progression of donor-derived disease/early CAV and thus serve as a marker of CAV. Indeed, this paradoxical association does not imply causation and has been consistently observed in prior SRTR-based studies.^5^

## Conclusions

PPCM HT recipients did not have a higher risk of developing midterm angiographic CAV compared with NIDCM recipients in the contemporary era. However, these data should be interpreted with caution given the aforementioned limitations and do not exclude the possibility of between-group differences emerging over time; as such, future studies with long-term follow-up are required to assess the durability of these findings.

## Funding support and author disclosures

The data reported here have been provided by the Hennepin Healthcare Research Institute (HHRI) as the contractor for the Scientific Registry of Transplant Recipients (SRTR). The interpretation and reporting of these data are the responsibility of the author(s) and in no way should be seen as an official policy of or interpretation by the SRTR or the U.S. Government. Dr Parikh has received research support from Abbott Vascular, Bayer, and Infraredx (Nipro); and consulting fees from Abbott Vascular, Cath-Works, and Nipro. All other authors have reported that they have no relationships relevant to the contents of this paper to disclose.
